# The 5-HT1A receptor agonist buspirone improves esophageal motor function and symptoms in systemic sclerosis: a 4-week, open-label trial

**DOI:** 10.1186/s13075-016-1094-y

**Published:** 2016-09-01

**Authors:** George P. Karamanolis, Stylianos Panopoulos, Konstantinos Denaxas, Anastasios Karlaftis, Alexandra Zorbala, Dimitrios Kamberoglou, Spiros D. Ladas, Petros P. Sfikakis

**Affiliations:** 1 Academic Department of Gastroenterology, “Laiko” Hospital, Athens Medical School National and Kapodistrian University, Athens, Greece; 2 Joint Academic Rheumatology Programme, Athens Medical School National and Kapodistrian University, Athens, Greece

**Keywords:** Scleroderma, Buspirone, Esophageal motility, Reflux symptoms

## Abstract

**Background:**

Acute administration of the oral 5-HT_1_A receptor agonist buspirone, which is commonly used as an anxiolytic drug, may improve compromised lower esophageal sphincter function. In an open-label trial we assessed the effects of buspirone on esophageal motor function and symptoms in patients with esophageal involvement associated with systemic sclerosis (SSc).

**Methods:**

Thirty consecutive patients with SSc and symptomatic esophageal involvement, despite treatment with proton pump inhibitors, underwent high resolution manometry and chest computed tomography for assessment of motor function and esophageal dilatation, respectively. Regurgitation, heartburn, dysphagia, and chest pain severity was subjectively scored by visual analog scales. Manometric parameters (primary endpoint) and symptom severity (secondary endpoint) were re-examined after 4-week daily administration of 20 mg buspirone. Other medications remained unchanged.

**Results:**

Eight patients did not complete the trial because of buspirone-associated dizziness (*n* = 2), or nausea (*n* = 2), or reluctancy to undergo final manometry. In the remaining 22 patients lower esophageal sphincter (LES) resting pressure increased from 7.7 ± 3.9 to 12.2 ± 4.6 mmHg (*p* = 0.00002) after buspirone administration; other manometric parameters did not change. Statistical analysis revealed negative correlation between individual increases in resting LES pressure and supra-aortic esophageal diameter (*r* = -0.589, *p* = 0.017), suggesting a more beneficial effect in patients with less severely affected esophageal function. Heartburn and regurgitation scores decreased at 4 weeks compared to baseline (*p* = 0.001, and *p* = 0.022, respectively).

**Conclusion:**

Our findings warrant more conclusive evaluation with a double-blind controlled study; however, buspirone could potentially be given under observation for objective improvement in all patients with SSc who report reflux symptoms despite undergoing standard treatment.

**Trial registration:**

ClinicalTrials.gov Identifier: NCT02363478 Registered: 21-02-2014.

## Background

Systemic sclerosis (SSc) is a chronic autoimmune fibrotic disease affecting the skin and other organs including the gastrointestinal tract. The esophagus is commonly affected in SSc and esophageal function is compromised in up to 90 % of patients [1, 2]. Symptoms of esophageal disease are due to gastroesophageal reflux disease (GERD) and esophageal motor dysfunction. Thus, heartburn, regurgitation and dysphagia have been reported by 80 % of patients with SSc [3, 4].

Although several medications are used in patients with symptomatic SSc and esophageal involvement, there is no universally effective treatment. The current therapeutic approach includes administration of proton pump inhibitors (PPIs) and prokinetics agents (including metoclopromide, erythromycin, cisapride and domperidone), although the evidence to support their use is limited [5]. It is well-known that there are many issues in the efficacy and long-term use of the currently available prokinetic drugs; use of metoclopromide or erythromycin or cisapride is restricted due to safety profile issues and administration route problems, while administration of domperidone has no effect on esophageal motor dysfunction in patients with symptomatic SSc [6].

Animal studies have identified serotonin (5-hydroxytryptamine, 5-HT) as one of the putative neurotransmitters involved in esophageal motor function making its receptors suitable therapeutic targets [7]. Studies in healthy volunteers show that acute administration of buspirone, a 5-HT_1_A receptor agonist, has a strong stimulatory effect on esophageal peristalsis and lower esophageal sphincter (LES) function [8, 9]. Moreover, we have recently observed that acute administration of buspirone exerts a beneficial effect on esophageal motor dysfunction associated with SSc [6]. Thus, in order to investigate whether buspirone exerts a long-term effect in SSc-associated esophageal involvement we conducted a prospective 4-week open-label trial. The primary objective was to assess the efficacy of buspirone on LES dysfunction. The secondary efficacy endpoint was change in esophageal symptoms after buspirone administration.

## Methods

### Study population

Thirty consecutive consenting patients who fulfilled classification criteria for SSc [10] and were able to perform high resolution manometry (HRM) participated in this open-label, non-randomized study. The only inclusion criterion was the presence of symptoms associated with esophageal involvement, such as regurgitation, heartburn, dysphagia, and chest pain, despite standard treatment with PPIs. These symptoms were scored on a 100-point visual analog scale (VAS) ranging from 0 (absent) to 100 (very severe)..Other types of esophageal diseases that could explain patients’ symptoms were excluded because all patients had undergone gastroscopy in the past.

Eight patients did not complete the study; 4 patients reported adverse effects with buspirone administration (2 dizziness and 2 nausea) and 4 patients were reluctant to perform the second HRM examination. The remaining 22 patients (aged 52.5 ± 11.6 years, 19 women) were re-examined after 4 weeks. During the study period all patients continued their therapy unchanged, including administration of proton pump inhibitors. All patients were on single PPIs dose before and during the study period and none was on medications that could affect results, such as prokinetics or erythromycin.

Before proceeding to esophageal manometry studies all patients filled out a self-reported symptom questionnaire in order to assess the severity of esophageal symptoms. Severity of dysphagia, heartburn, regurgitation, and chest pain was measured on a VAS (0–100). The VAS score has been adopted in many other trials for evaluation of visceral symptoms and has been used as a tool for self-assessment of symptoms [11, 12], and any decrease in the score at week 4 versus baseline was considered an improvement. Demographic data and phenotypic characteristics of the disease were collected from the clinical charts. Moreover, in all patients the supra-aortic and infra-aortic coronal diameters of the esophagus were measured in chest computed tomography (CT) performed within the previous 3 months. Esophageal dilatation was deemed present when the diameter was >9 mm [13]. The study protocol was approved by the ethics committee of “Laikon” General Hospital and informed consent was obtained from all participating patients.

### High resolution manometry

All patients were fasted and were studied in the supine position. Esophageal manometry was performed with a water-perfused assembly with 22 pressure sensors (Solar GI HRM, MMS, Enschede, The Netherlands). The HRM catheter was passed trans-nasally and positioned to record from the hypopharynx to the stomach. The catheter was fixed in place by taping it to the nose. The manometric examination included a 30-sec period to assess basal LES pressure and 10 swallows of 5 mL water. Double swallows were discarded and these were repeated. The study was repeated 4 weeks after oral administration of 20 mg buspirone (10 mg twice a day). We recorded 1) amplitude, duration and velocity of contractions at the distal part of the esophagus (defined as the mean amplitude at 3 and 8 cm proximal to the LES upper border of the esophagus) and 2) resting and residual LES pressure and integrated relaxation pressure (IRP). The LES pressures were referenced to intragastric pressure and we reported the mean resting pressure values.

All HRM tracings were interpreted by the same investigator (GK), who was blinded to whether administration of buspirone was performed. Hypotensive LES was considered when LES resting pressure was ≤10 mmHg, whereas isobaric contour <20 mmHg was considered as the cutoff for esophageal hypomotility [14].

### Statistical methods

The paired-sample *t* test was used to compare changes from baseline in manometric values and severity of symptoms after buspirone administration, as all data analyzed were normally distributed. The Spearman correlation coefficient was used to assess the relationship between increase in LES pressure and other clinical, manometric, and CT parameters, because of non-canonical data distribution. Values were expressed as mean ± SD. A *p* value <0.05 was considered significant.

## Results

Table 1 summarizes demographic characteristics, expression and duration of disease, CT, baseline manometry, and severity of esophageal symptoms. The mean amplitude of distal esophageal contractions was 16.6 ± 9.6 mmHg, whereas esophageal hypomotility was identified in 19 patients (86 %). The mean resting pressure of the LES was 7.7 ± 3.9 mmHg, whereas a hypotensive LES was observed in 17 patients (77 %). Both esophageal body and LES abnormalities were found in 16 patients (73 %). According to the Chicago classification 16 patients had absent peristalsis, 5 patients had weak peristalsis and 1 patient had normal esophageal motility. On chest CT 12 patients (54.5 %) had supra-aortic esophageal dilatation, whereas almost all patients had infra-aortic esophageal dilatation (21/22, 95.5 %).

**Table 1:** Demographic characteristics, disease characteristics, symptoms, and manometry in the study population (*n* = 22)

Variable	Value
Age (years)	52. 5 ± 11.9
Sex (female/male)	19/3
Duration of disease (years)^a^	8.7 ± 6.7
Diffuse SSc (*n* (%))	16 (72.7)
Pulmonary fibrosis^b^	15 (68.1)
Digital ulcers^c^	10 (45.5)
Anti-Scl 70 (%)	9 (41 %)
ANA (%)	19 (86.4 %)
ACA (%)	2 (9.1 %)
CPK (> × 2 normal values) (%)	2 (9.1 %)^d^
Supra-aortic coronal diameters (cm)	12.4 ± 5.3
Infra-aortic coronal diameters (cm)	22.5 ± 11.1
Frequency of symptoms (%)	
-dysphagia	12 (54.5)
-heartburn	20 (91.0)
-regurgitation	19 (86.0)
-chest pain	8 (36.4)
severity of symptoms (0–100)	
-dysphagia	25.8 ± 30.7
-heartburn	27.1 ± 24.0
-regurgitation	39.3 ± 29.8
-chest pain	10.4 ± 2.1
amplitude of distal contractions (mmHg)	16.6 ± 9.6
duration of distal contractions (cm)	3.7 ± 2.4
velocity of distal contractions (cm/sec)	2.6 ± 1.9
LES resting pressure (mmHg)	7.7 ± 3.9
LES residual pressure (mmHg)	2.9 ± 1.9
IRP (mmHg)	3.1 ± 2.3

*SSc* systemic sclerosis, *ANA* antinuclear antibody, *ACA* anti-centromere antibody, *LES* lower esophageal sphincter, *IRP* integrated relaxation pressure, *HRCT*, High resolution computed tomography, *CPK*, creatinophosphokinase, *anti-Scl*, anti-topoisomerase I

^a^Disease duration was from first non-Raunaud’s symptom

^b^Presence of interstitial lung disease was determined after evaluation of HRCT

^c^Five out of these ten patients had active digital ulcers at the time of the manometry

^d^None of the patients had clinical signs of myositis or skeletal myopathy

Heartburn and regurgitation were reported by almost all patients (91 % and 86 %, respectively), while dysphagia was reported by 54.5 % of patients. Only 36 % of patients reported chest pain as one of their symptoms. Moreover, regurgitation and heartburn were the most bothersome symptoms reported to a similar extent, followed by dysphagia and chest pain (Table 1).

### Effect of buspirone on manometric parameters

**Fig. 1:**
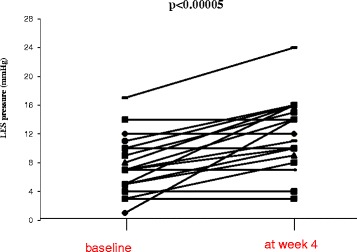
Change in lower esophageal sphincter (*LES*) pressure in each individual at baseline and after 4-week daily oral administration of 20 mg buspirone

Following buspirone administration for 4 weeks the LES resting pressure significantly increased from 7.7 ± 3.9 to 12.2 ± 4.6 mmHg (*p* < 0.00005). Figure 1 increased LES resting pressure after buspirone administration was observed in 15 patients (68 %) with a range of 4–13 mmHg (41–220 %, accordingly). There were significantly fewer patients with hypotensive LES after compared to before buspirone administration (8/22 vs. 17/22, respectively, *p* = 0.006).

In contrast to LES resting pressure, buspirone administration had no effect on LES residual pressure or on IRP (Table 2). In addition, buspirone administration did not increase the duration and velocity of esophageal body contractions or the amplitude of contractions, compared to baseline values (Table 2). Buspirone was well-tolerated by all patients who completed the study.

**Table 2:** Manometric parameters at baseline and after 4-week daily administration of 20 mg buspirone

	Baseline	After buspirone	*p*
Amplitude (mmHg)	16.6 ± 9.6	17.5 ± 9.6	0.163
Velocity (cm/sec)	3.7 ± 2.4	3.4 ± 1.9	0.701
Duration (sec)	2.6 ± 1.9	3.1 ± 2.3	0.668
LES resting pressure (mmHg)	7.7 ± 3.9	12.2 ± 4.6	0.00002
LES residual pressure (mmHg)	2.9 ± 1.9	3.2 ± 2.1	0.157
IRP (mmHg)	3.1 ± 2.3	3.5 ± 2.1	0.143

*LES* lower esophageal sphincter, *IRP* integrated relaxation pressure

### Correlation between individual increase in LES pressure and other clinical, manometric and CT parameters

Table 3 summarizes the correlation between individual increase in LES pressure and other variables. There was moderate, but significant, inverse correlation between increased LES resting pressure and supra-aortic diameter (*p* = 0.017). The Pearson correlation coefficient was an r value of -0.589. In patients without supra-esophageal dilatation there was greater improvement in LES pressure (83.9 ± 38.1 % vs.19.2 ± 26.8 %, *p* = 0.037). There was no significant correlation between increased LES pressure and other measured parameters.

**Table 3:** Correlation between increase in LES pressure after 4-week daily administration of 20 mg buspirone and clinical, manometric and computed tomography parameters

	*r*	*p*
Baseline LES	-0.356	0.113
Supra-aortic diameter	-0,589	0.017
Infra- aortic diameter	-0.406	0.191
Disease duration	-0.226	0.325
Heartburn	0.129	0.578
Regurgitation	-0.188	0.415
Chest pain	0.103	0.657
Dysphagia	0.188	0.414

*r* Spearman correlation coefficient, *LES* lower esophageal sphincter

### Effect of buspirone on esophageal symptoms

The severity of regurgitation and heartburn significantly decreased from baseline following buspirone administration for 4 weeks (39.3 ± 29.8 vs. 24.4 ± 22.0, *p* = 0.02 and 37.1 ± 23.9 vs. 21.9 ± 21.2, *p* = 0.001, respectively). There was no significant improvement in the scores for severity of chest pain and dysphagia (10.4 ± 23.1 vs. 8.5 ± 20.0 and 25.8 ± 30.7 vs. 18.9 ± 21.9, *p* = 0.203, respectively).

Improvement in the severity of heartburn was reported by 70 % (14/20) of patients who had heartburn at baseline, while 58 % (11/19) of patients with regurgitation at baseline had improvement in the severity of regurgitation. Increased LES resting pressure was simultaneously observed in the majority of patients with improvement in heartburn and regurgitation (12/14 and 8/11, respectively). Half of the patients with baseline dysphagia reported improvement in their symptom severity, while a minority of patients (25 %) with baseline chest pain had an improvement in the severity of their symptoms.

## Discussion

Treatment of patients with symptomatic SSc and esophageal involvement is still an area of unmet need, as there is no available treatment with established efficacy. Administration of PPIs and prokinetic drugs do not appear to provide substantial symptomatic relief in patients with SSc and esophageal symptoms [5]. Pathophysiology of gastrointestinal dysmotility in SSc is multifactorial and different neurotransmitters could be involved in this process [15, 16]. Serotonin (5-HT) is considered a key neurotransmitter and acute studies, both in healthy subjects and patients with SSc, have shown that agonists of specific class of its receptors, such as 5-HT_1_A, could be a putative therapeutic option [6, 8, 9]. Buspirone had been initially developed for clinical use in the treatment of depression and anxiety disorders. Its mechanisms of action are thought to include agonistic actions, mainly on 5-HT_1_A receptors, but also on dopamine D2 receptors. Buspirone is absorbed rapidly and almost completely with a t_max_ = 0.89 ± 0.15 h [17].

To the best of our knowledge no previous study has prospectively investigated whether a pharmaceutical agent may help patients with SSc and esophageal involvement. The results of our open-label, prospective 4-week trial suggest that the 5-HT_1_A receptor agonist, buspirone, given orally for 4 weeks, significantly increases the LES resting pressure by 41 % to 220 % over the baseline values in almost 70 % of patients with SSc. Furthermore, patients with a less dilated esophagus as detected on chest CT had a better response to buspirone administration. This is an important aspect of our study, as patients with SSc undergo routine chest CT as a screening test for lung fibrosis. Many studies have suggested that measurement of esophageal coronal diameter on chest CT is a marker of the functional status of the esophagus in SSc and a dilated esophagus represents more severe visceral involvement [18, 19].

Herein, the 4-week administration of buspirone also alleviated heartburn and regurgitation in the majority of patients. Buspirone seems more effective for GERD-related symptoms, whereas symptoms related to esophageal hypomotility, such as dysphagia and chest pain are not affected. Thus, we could suggest that buspirone administration may be most helpful for the subgroup of SSc patients with reflux symptoms. An objective assessment of reflux response with pH-impedance measurement could certainly enhance our results.

As all patients were on PPIs before and during the study period, the strategy with add-on buspirone could be a worthwhile therapeutic option. Keeping in mind the anecdotal evidence that persistent reflux contributes significantly to the development of interstitial lung disease and/or progress, which is the leading cause of death in SSc, the potential beneficial effect of buspirone becomes more important. Buspirone-associated improvement in the severity of reflux symptoms can be explained by a strong stimulatory effect on LES resting pressure. However, other mechanisms cannot be excluded. Comorbid psychiatric diseases in patients with a chronic disorder, such as SSc, and especially anxiety, are common [20, 21]. Buspirone is used as anxiolytic drug in clinical practice. Therefore, a potential alteration in the sensitivity by decreasing the anxiety level may play a role in symptom improvement. As we did not include measurement of anxiety scores, this is a limitation of the present study. However, in the event that the main mechanism underlying symptom improvement with buspirone was the anxiolytic effect of the drug, other non-reflux symptoms should have been improved.

A limitation of the present study, due to restrictive regulatory rules that we were unable to overcome, is that it is not a randomized, placebo-controlled investigation and there is a lack of a control arm. Another limitation is the relatively small number of patients studied; however, we have to keep in mind that patients with SSc due to the chronic nature of their disease have several medical disabilities, making recruitment very challenging. The use of a validated questionnaire, such as the UCLA STCC SSc-GI [22] rather than a self-reported one could strengthen our results, however, this questionnaire, which covers a broad spectrum of gastrointestinal symptoms in patients with SSc, is not validated in Greek. Moreover, the majority of available questionnaires are focused on reflux symptoms rather than on dysmotility-associated symptoms characterizing SSc.

Buspirone was well-tolerated in the present study. Treatment interruption due to adverse effects (dizziness and nausea) was observed in only 4 out of 30 patients. Patients who completed the study reported sporadic and self-limited adverse effects that did not affect their daily activities.

## Conclusions

In conclusion, our study showed that 4-week daily administration of buspirone increases the LES resting pressure and improves esophageal symptoms in the majority of SSc patients with esophageal involvement. Furthermore, buspirone exerts a greater beneficial effect on patients with less severely affected esophageal function as detected on chest CT, suggesting a therapeutic role mainly in patients with earlier or less severe involvement. Although further evaluation in placebo-controlled studies is warranted, buspirone could potentially be given under observation for objective improvement in all patients with SSc who report reflux symptoms, despite undergoing standard treatment.

## Abbreviations

5-HT: 5-hydroxytryptamine
CT: computed tomography
GERD: gastroesophageal reflux disease
HRM: high resolution manometry
IRP: integrated relaxation pressure
LES: lower esophageal sphincter
SSc: systemic sclerosis
VAS: visual analog scale
